# Schoolchildren’s Compensatory Strategies and Skills in Relation to Attention and Executive Function App Training

**DOI:** 10.3389/fpsyg.2019.02332

**Published:** 2019-10-15

**Authors:** Teresa Rossignoli-Palomeque, María Quiros-Godoy, Elena Perez-Hernandez, Javier González-Marqués

**Affiliations:** ^1^Department of Basic Psychology II, Complutense University of Madrid, Madrid, Spain; ^2^Department of Psychology and Education, Cardenal Cisneros University Center, Madrid, Spain; ^3^Department of Social Psychology and Methodology, Autonomous University of Madrid, Madrid, Spain; ^4^Department of Development and Educational Psychology, Autonomous University of Madrid, Madrid, Spain

**Keywords:** inhibition, vigilance, procedural metacognition, application, children, attention, executive functions, cognitive training

## Abstract

**Background:**

Given the importance of attention and executive functions (EF) in children’s behavior, programs aimed at improving these processes are of special interest. Nexxo-training combines the use of the Nexxo touchscreen application (inhibition and vigilance tasks) with procedural metacognitive strategies (imparted by an instructor) for all the individuals using the app, regardless of their level of ability, plus compensatory strategies based on individual child performance. This study presents an analysis of the compensatory strategies that schoolchildren (aged 6–8 years old) receive when experiencing difficulties with EF tasks, in addition to an analysis of the developmental factors and cognitive skills that may modulate EF task performance.

**Methods:**

For this study, we use data from a previous randomized active-controlled study (under review), in which forty-six typically developing children aged between 6 and 8 years old (24 girls/22 boys) were enrolled in the training group. The selected children were in the 1st grade (*n* = 28, $\bar{x}$ = 78.32 ± 4.037 months) and 3rd grade of primary education (*n* = 18, $\bar{x}$ = 102.11 ± 3.445). We collected data on EF training performance, compensatory strategies needed and neuropsychological assessments.

**Results:**

A total of 80.43% participants required some form of compensatory strategy during training. Regarding required compensatory strategies, those who had lower scores in EF training needed more compensatory strategies, in particular, instructional comprehension (*r* = −0.561, *p* < 0.001 for inhibition-tasks; *r* = −0.342, *p* < 0.001 for vigilance-tasks). Concerning developmental factors, age significantly predicted better performance in both EF tasks (β = 0.613, *p* < 0.001 for inhibition; β = 0.706, *p* < 0.001 for attention). As regards task performance, those with better performance in inhibition tasks also had better performance in vigilance tasks (*r* = 0.72, *p* < 0.001). Finally, regarding cognitive skills, participants with higher performance in fluid intelligence (Q1, *n* = 12) had higher scores (*U* = 14.5, *p* < 0.05) than the group with the lowest performance (Q4, *n* = 11) in vigilance.

**Conclusion:**

As previous literature suggests, inhibition is one of the core processes of EF. Therefore, we should focus training on the core EF processes. Inhibition and vigilance are closely related processes. In terms of the use of compensatory strategies, these are more needed for participants with lower levels of performance in inhibition or vigilance. Regarding strategy analysis, instructional comprehension and self-instruction (goal setting and planning) seem to be the most useful strategies for those with difficulties in inhibitory and vigilance task performance. Regarding development, as expected, age moderates task performance in inhibition and attention. Finally, cognitive skills, such as fluid intelligence and cognitive flexibility, predicted better results in attention. EF training using not only an app, but also compensatory strategies based on user performance, is a new research direction offering more opportunities to generalize EF training in everyday life.

## Introduction

Executive Functions (EF) can be understood as a variety of interrelated processes that help to direct and control mental abilities to accomplish a task or goal (Reck and Hund, 2011). Miyake et al. (2000) propose a hierarchical model in which EF is considered as a unitary construct with three main components: (1) inhibition, (2) updating, and (3) shifting. Inhibition is the ability to suppress one automatic or prepotent response in favor of another, or to suppress the response altogether, known as response inhibition. Another aspect of inhibition is interference control, which is required to select relevant stimuli when a distractor appears (Miyake et al., 2000; Diamond, 2013; van der Ven et al., 2013; Tamm and Nakonezny, 2015). This process is one of the first stages to develop and is thought to be responsible for changes in other EF components (Dempster, 1992; Gandolfi et al., 2014). Updating is the ability to retain and manipulate information during a short period of time (Miyake et al., 2000; Klingberg et al., 2002). This ability is essential for learning (Conway et al., 2003). Finally, shifting is the ability to change from “one mental set” to another (Miyake et al., 2000). These components are involved in several everyday activities (Diamond, 2013).

Previous studies have found a relation between EF and intelligence (Andersson, 2008; Molfese et al., 2010; Karbach and Unger, 2014); however, EF is even more predictive of academic success than IQ (Gathercole et al., 2004; Blair and Razza, 2007). Apart from academic success, EF also seems to have an impact on social adjustment (Bryck and Fisher, 2012). “Social adjustment is defined as the degree to which children get along with their peers; the degree to which they engage in adaptive, competent social behavior; and the extent to which they inhibit aversive, incompetent behavior” (Crick and Dodge, 1994, p.82). Difficulties in EF are present in social maladjustment (Olson, 1989; Blair and Razza, 2007). EF components are impaired in various childhood disorders (Barkley, 1997), such as ADHD (Rebollo and Montiel, 2006; Gau et al., 2010), autism (Ciesielski and Harris, 1997), obsessive-compulsive disorder (Enright and Beech, 1993), and behavioral disorders (Rebollo and Montiel, 2006). For these reasons, studies on EF interventions in children and the mechanisms involved in their development are relevant. This knowledge can be applied to EF programs aimed at school settings for typically developing children as a protective factor or in clinical contexts for those with EF difficulties as part of the intervention.

If inhibition is one the core components of EF, the intensity domain of attention is the core component of attention (Sturm, 2008). The intensity domain involves alertness, sustained attention and vigilance as the basis of attention (Hauke et al., 2011). Tonic alertness is thought of as a top-down control function of the arousal system without the influence of external stimuli, whereas phasic alertness is the capability to respond following a warning stimulus (Sturm and Willmes, 2001). Sustained attention involves the detection of changes over a long period with a high rate of relevant stimuli. In contrast, vigilance, a state of sustained alertness, involves the detection of changes when only a low rate of relevant stimuli exists (Hauke et al., 2011). Some aspects of attention overlap with certain components of EF (Rueda et al., 2012), which explains the high degree of interaction between attention and EF. The core processes of attention and EF are related; for instance, inhibition is fundamental for attentional maintenance (Pontifex et al., 2012). Furthermore, previous research has found that children with higher levels of sustained attention present high levels of inhibitory control (Reck and Hund, 2011). Sustained attention and behavioral inhibition interact throughout child development. A longitudinal study (testing attention at 9 months and studying behavioral inhibition until adolescence) demonstrated that sustained attention is related to inhibitory control. Individuals with lower levels of sustained attention presented increased levels of behavioral inhibition during childhood and social discomfort during adolescence (Pérez-Edgar et al., 2010). Apart from sustained attention, vigilance and inhibitory control are closely related (Lovejoy and Rasmussen, 1990).

Studying the attentional element involved in EF tasks, procedural metacognitive strategies (including self-regulatory strategies) and related skills may help us to design EF training strategies and interventions based on scientific data. Attention is strongly needed in EF tasks, and EF and self-regulation share resources (Kaplan and Berman, 2010). Some attention training has shown benefits in EF tasks. One study demonstrated how attention training in children with ADHD not only reduced symptoms of inattentiveness, but also enhanced EF, specifically, by shifting attention (La Marca and O’Connor, 2016). Studies on attention span and working memory have shown how training benefits participants with ADHD with regard to EF (Klingberg et al., 2002, 2005; Beck et al., 2010). In our view, due to the interaction between attention, EF and self-regulation, training that combines these processes may produce more transfer effects than just training EF alone. Following this hypothesis, our team developed Nexxo-training, which aims to improve vigilance, inhibition and procedural metacognitive strategies in typically developing children.

Most cognitive training can be classified into two categories: process-based training and strategy-based training (Morrison and Chein, 2011; Jolles and Crone, 2012). Both approaches involve practice or intentional instruction to improve cognitive skills. The main difference is that strategy-based training uses more explicit task instructions than process-based training (Jolles and Crone, 2012). Regarding attention and EF training, a few process-based training methods have shown positive effects in typically developing children, either in terms of attention (Thorell et al., 2009) executive attention (Rueda et al., 2005), fluid intelligence (Klingberg et al., 2005; Liu et al., 2015), or academic performance (Dahlin, 2011, 2013; Holmes and Gathercole, 2014). Nevertheless, the limitations of process-based training have been found in the far transfer or generalization of the training in the user’s everyday life. Similarly, limitations have been found in long-term effects (Rossignoli-Palomeque et al., 2018). The aim of EF training should be the generalization of the training in children’s daily life, in cognitive skills, academic performance, and social adjustment, which are considered “far transfer.” A significant number of previous studies on EF training efficacy fail to find or examine these types of transfer results (Rossignoli-Palomeque et al., 2018). To overcome this limitation of traditional process-based training, strategy-based training provides guidance with the tasks which help users to identify the strategies needed to perform those tasks. An example of this kind of guidance is scaffolding, or metacognitive strategies, designed in combination with the training (Pozuelos et al., 2018). Indeed, strategy-based training has yielded positive results. Pozuelos et al. (2018) compared two groups with executive attention training in typically developing children with an active control group. One of the training groups followed traditional attention and EF protocol, whereas the other underwent metacognitive strategies. The children in the metacognitive group showed not only greater gains in intelligence, but also significant increases in conflict processing, measured through electrophysiological techniques. In addition, changes in brain activity regarding conflict processing predicted gains in intelligence in this group. The EF and attention intervention program that we analyze, called Nexxo-training, combines inhibition and vigilance training through a touchscreen application with strategies of “procedural metacognition” directed by a single instructor. This strategy-based training consists of repeating a task in combination with strategies to improve performance tasks. The unique feature of this specific strategy-based training is that the training provides not only procedural metacognitive strategies (i.e., general strategies for the whole group), but also compensatory strategies for participants who experience greater difficulty during the training. In this way, the developmental processes involved in the attention and EF training task can be easily improved and generalized. A previous study of Nexxo-training, a randomized-controlled study, showed far transfer after training in supervision, attention and EF as reported by parents (Rossignoli-Palomeque et al., submitted). Far transfer occurs when training effects are produced in tasks or constructs that have not been directly trained. By contrast, near transfer occurs when the effects are reflected in similar tasks to those that have been directly trained (Karbach and Unger, 2014). Further research on this type of training is crucial as it offers a new direction for cognitive training interventions.

In addition, to plan any form of attention and EF intervention, developmental factors must also be considered. In general, the initial manifestations of EF occur during the 1st year of life, with accelerated development in childhood (Carlson and White, 2013). EF development may be a pyramidal process. Certain basic components, such as inhibition, will later support the development of other more complex processes, such as flexibility (Flores-Lázaro et al., 2014). Nevertheless, other components, such as planning, do not reach adult levels until approximately the age of 12 years old while others, such as abstraction, will continue to develop into adulthood (Zelazo and Müller, 2002) reaching peak performance at around 20–30 years of age (Blakemore and Choudhury, 2006). Regarding attention, conscious control of attention increases between 2 and 6 years of age (Rothbart and Posner, 2001; Diamond et al., 2007). There is a second significant improvement in cognitive control of attention at around 9–12 years of age (Pozuelos et al., 2014). Meanwhile, sustained attention improves significantly between the ages of 3 and 5 years old (Garon et al., 2008) and continues to develop progressively throughout a child’s school years. There are significant changes in sustained attention from 6 to 7 years of age in comparison with 10- to 11-year-olds (Lewis et al., 2017b). Inhibition and attention are relevant cognitive abilities. In terms of development, go/no-go tasks have demonstrated a significant improvement in response inhibition and sustained attention between the ages of 6 and 8 years old, while these changes are more subtle from 8 to 11 years of age (Lewis et al., 2017a). Previous studies, using go/no-go tasks for assessment, support the same idea that there is an improvement in response inhibition abilities between the ages of 6 and 8 years (Becker et al., 1987). Inhibition is a process that develops particularly between the ages of 5 and 10 years (Urben et al., 2011).

Apart from the relation between attention, EF and developmental factors, it is also worth considering what other skills and strategies may be involved in performing attention and EF tasks successfully. Previous studies have shown that inhibition training in preschoolers produced a trend-level improvement in reasoning and neural changes in the experimental group (Liu et al., 2015). Other authors suggest that students with a high IQ also perform well in EF tasks, specifically in inhibition and flexibility (Sastre-Riba and Viana-Sáenz, 2016). On the other hand, lower vigilance performance has been linked to a lower IQ in children who are at risk of learning disabilities (Swanson and Cooney, 1989). Therefore, if attention, EF and intelligence are related, which specific cognitive abilities are involved, and which are better at predicting attention and EF performance? These crucial questions must be addressed by attention and EF training developers.

Regarding schoolchildren’s use of procedural metacognitive strategies in inhibitory tasks, it seems that verbal strategies (e.g., verbalizations of what to do/not do) and motor strategies (e.g., moving away, shaking their heads, covering their mouths, etc.) are used by preschoolers to inhibit themselves (Fatzer and Roebers, 2013). The combination of both types of strategies seems to produce better inhibitory results (Manfra et al., 2014). The development of these strategies depends on the child’s age. For instance, verbalizations and inner speech evolve between 2 and 8 years of age, from irrelevant speech to self-directed verbalizations, both of which are relevant to the task (Winsler et al., 2009). Another type of strategy, which seems to promote better results in EF tasks in older students and adults, are self-instructions (e.g., saying out loud what to do, how to do it, etc.) (Karbach and Kray, 2009). The development of these strategies varies throughout child development (Vygotsky et al., 1978; Bjorklund and Harnishfeger, 1990) and is also based on the level of task difficulty (Fernyhough and Fradley, 2005). Nexxo-training strategies consist of procedural metacognitive strategies. These strategies involve self-regulation (motor and verbal strategies), instructional comprehension, and self-instruction strategies, according to the participant’s development. Self-instruction and instructional comprehension involve three phases: (1) forethought (establish goals, “what do I have to do?”), (2) performance/volitional control (planning, monitoring and controlling cognition, “how am I going to do it?”) and, (3) self-reflection (self-evaluation and cognitive flexibility to make adjustments if required). These three phases are metacognitive strategies that can be applied in self-regulated learning (Dina and Efklides, 2009). EF and procedural metacognition (such as the strategies mentioned above) share common theoretical characteristics, developmental paths, and even brain regions. Therefore, the student’s control of their own learning is crucial (Roebers and Feurer, 2016). To our knowledge, this is the first EF training that offers these strategies for school-aged students. The primary focus of this study was to analyze the strategies that students (aged 6–8 years old) use when confronted with challenging strategy-based EF and attention training (“Nexxo-training”). This training, delivered through an online application, combines inhibition and vigilance training with procedural metacognitive strategies. The study also analyzes the cognitive skills and developmental factors that may modulate task performance.

The study objectives are as follows: (1) to determine whether procedural metacognitive strategies have an impact on task performance and which ones are relevant; (2) to ascertain whether age moderates the use of strategies and task performance; (3) to identify which cognitive skills are related to task performance as possible predictors; and (4) if cognitive skills are predictive of task performance, the final objective is to test whether this relation is sustainable when the lowest and highest levels of performance are compared.

This information is crucial to the scientific development of new training technologies for EF and attention interventions.

## Materials and Methods

### Ethics Statement

In accordance with the Declaration of Helsinki, written informed consent was obtained from each parent’s participant. This study was approved by the ethics committee of the San Carlos Hospital (n° 15/315-E) in June 2015.

### Participants

The study participants were recruited from two schools after receiving their parents’ consent. Forty-six typically developing children aged between 6 and 8 years old (24 girls and 22 boys) participated in the study. The selected children were in the 1st grade (*n* = 28, $\bar{x}$ = 78.32 ± 4.037 months) or 3rd grade of primary education (*n* = 18, $\bar{x}$ = 102.11 ± 3.445). The parents’ average professional range was $\bar{x}$ = 2.59 ± 0.53 (0 = low level, 1 = medium-low, 2 = medium, 3 = medium-high, and 4 = high) according to the “National Institute of Professional Range” (Spain). The inclusion criteria were as follows: (1) between the ages of 5–7 and 8–9 years; (2) no previous diagnosis of diseases or disorders related to developmental delays; (3) no psychological or speech therapy treatment required at the time of the study or earlier; (4) Spanish-speaking (monolingual); and (5) no diagnosis of learning difficulties or repetition of school year. Criteria 1–5 were obtained through a parents’ questionnaire. Table 1 shows the sociodemographic description of the participants.

**TABLE 1:** Sociodemographic description of participants.

	**Female (*n* = 24)**				**Male (*n* = 22)**				**Total (*n* = 46)**			
	**Mean**	***SD***	**Min**	**Max**	**Mean**	***SD***	**Min**	**Max**	**Mean**	***SD***	**Min**	**Max**
Age	7.04	1.06	6	9	6.62	0.973	5	8	6.85	1.03	5	9
IQ	104	13.9	79	131	106	16.1	78	130	105	14.8	78	131

*SD = standard deviation; IQ = intelligence quotient measured by Reynold Intellectual Screening Test (RIST); Min = minimum; Max = maximum.*

### Assessments

#### Standardized Tests Were Used to Assess the Following Dimensions:

Cognitive skills through individual cognitive assessments (40–45 min): attention using the DIVISA-R “Trees Simple Visual Discrimination Test – Revised” (Santacreu et al., 2010), intelligence using the Reynolds Intellectual Screening Test (RIST) (Reynolds and Kamphaus, 2003), the Five Digit Test (FDT) (Sedó, 2007) to measure inhibition and cognitive flexibility, and, processing speed assessment through the Wechsler Intelligence Scale for Children-fourth edition (WISC-IV) (Wechsler, 2005).

The DIVISA-R (Santacreu et al., 2010) is a computer-based test in which the participant is required to tap the same trees as the model as quickly as possible. It takes approximately 15 min and is suitable for children aged 6–12 years. It provides five main indexes: distraction-precipitation, commission errors, omission errors, processing speed, and a global attention score. The reliability is based on Cronbach’s alpha ≥0.77 for all scales.

The RIST (Reynolds and Kamphaus, 2003) is a screening intelligence test. It contains two subscales: “guess what,” to assess verbal intelligence, and “odd-item-out,” to assess non-verbal intelligence. The sum of both subscales determines a general index of intelligence ($\bar{x}$ 100 ± 15). The reliability based on Cronbach’s alpha is 0.91.

The FDT (Sedó, 2007) is a test to measure certain aspects of EF (inhibition and cognitive flexibility). It contains four subscales: decoding, counting, election and alternative. It provides measures of inhibition and flexibility. In the inhibition subscale, the participant is required to count the numbers in a box instead of reading the numbers (automatic response). In the flexibility subscale, the participant must change strategy (from counting the numbers in a box to reading the number seen in the box), indicated by boxes in a blue frame. The Spearman-Brown coefficient ranges between 0.92 and 0.95.

The WISC-IV (Wechsler, 2005) implemented in this study included the Index of processing speed PSI (Coding and symbols searching). In coding, the participant is required to transcribe a digit-symbol code as quickly as possible for 2 min. In symbol searching, the participant is asked to decide whether target symbols appear in a row of symbols or not. These subscales were used to assess processing speed. The average internal consistency coefficient for PSI is 0.88.

Inhibition and vigilance through go/no-go and stop signal task performance: the Nexxo application provides a score of task performance for inhibition and vigilance for each session according to the number of errors (omissions and commissions) and successes. At the end of the training, the scores for each session in the different blocks are added up to obtain an overall score for the intervention, which is used to as a measure of task performance in inhibition and vigilance for each participant.

### Task

#### Go/No-Go and Stop Signal Tasks

The Nexxo application is based on neuropsychological models known as “go/no-go” and “stop signal” tasks (Shiffrin and Schneider, 1977; Logan, 1994), which involve a suppression of an ongoing response (inhibition), “n-back,” a typical task involving the temporary storage, manipulation, and selection of information (Tsujimoto et al., 2007) by deciding whether to make a response or not depending on whether a sequence is fulfilled (working memory), and, vigilance, in which changes are to be detected when only a low rate of relevant stimuli are presented (Sturm, 2008). As there is a low presence of these types of games (n-back) in level 1 of the Nexxo app (i.e., the one used in the study), we excluded them to focus on inhibition and vigilance processes. The game had two different blocks: vigilance vs. inhibition. In the vigilance block, the user had to tap the screen sporadically (differentiating between possible distractors and thus maintaining a state of alertness, also known as “vigilance”), whereas in the inhibition block, the user had to tap very frequently (holding back an automatic response, which is known as “inhibition or self-control”). The mechanics of the game included requirements to touch the screen when a specific stimulus was present, for example: “tap when you see that the figures on the screen are the same.” The screen turned green when the user tapped correctly and red when the user tapped incorrectly. The instructor applied compensatory strategies if the user displayed difficulties in carrying out the task.

**FIGURE 1:**
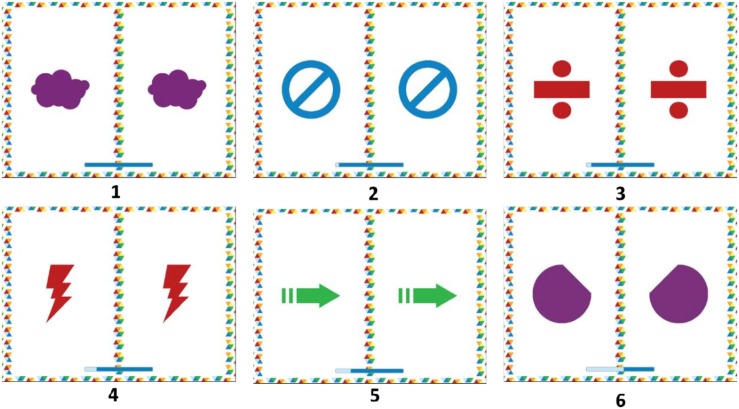
Nexxo activity example. **(2)** Screenshots of inhibition block. Instruction: “tap when you see that the figures on the screen are the same.” The user must tap all the screens except the last, where the hold response is required. Transitions between stimulus: 1000 ms. Nexxo 2016. Reproduced with permission of tapp-mobile. Number correspond with the order of stimulus appereance.

Figure 1 shows an example of a Nexxo activity.

Each game has a different command and stimulus presentation. In the vigilance block, the rate of target presence was less than 30% (70% no-go probability), whereas in the inhibition block the rate of target presence was over 70% (30% no-go probability). After each game, the participants were shown on the screen how many stars they had received as a reinforcement (0–3 depending on the level of performance). The participants played 30 games divided into two different blocks (15 vigilance games and 15 inhibition games) in the first level. There were 15 session in total (three games per session/each game was done twice) with each session lasting approximately 15 min. Additionally, Nexxo was developed to train processing speed (as the screen transition was set at one second, stimulus processing and the decision to tap or not tap required perceptual-motor agility). The Nexxo application also requires visual and auditory discrimination skills due to the presence of both types of stimuli in the form of targets and distractors (e.g., game V7 level 1 instruction: “tap each time you see a yellow circle with this sound”). Finally, Nexxo records the types of errors committed by the user: commission errors (the user tapped the screen when a response should have been withheld) and omission errors (the user did not tap when a response was required).

#### Procedural Metacognitive Strategies

The training also involved self-regulatory and self-monitoring strategies inspired by Perez-Hernandez and Capilla (2008), which were directed by the instructor and recorded for each participant in each session, as follows: (1) general instructions (for all participants): an instruction to get ready for the session (the participants had to put their hands over two fixed stickers when they heard “in position” and wait for the instructor to give further instructions), “visual self-instruction” (wait-see-tap), a visual reminder of how to perform the games in order to foster self-control, and verbal self-instructions: “I am a good observer, I do not fall into traps,” instructional comprehension/self-instruction (goal setting and planning): the instructor reads the instructions of the game out loud and asks the participants to say when and how they have to tap in each game though fixed questions (e.g., “when do we have to tap?” (the instructor) “we have to tap when…” (the participants) “how are we going to do it?” (the instructor) “we have to wait, see and tap”), and, verbal reinforcement after the games (e.g., “very good”); and (2) compensatory strategies (for participants who presented difficulties while performing the task): individual reinforcement if required (repeating the instruction to get ready, repeating self-instruction, repeating instructions, child verbalizations during the game (saying out loud what appears on the screen), or, in the latter case, instructor verbalizations (saying out loud what appears on the screen), and positive reinforcement through gestures (saying “well done” out loud).

More information about strategies applied can be seen in Supplementary Material.

### Procedures

**FIGURE 2:**
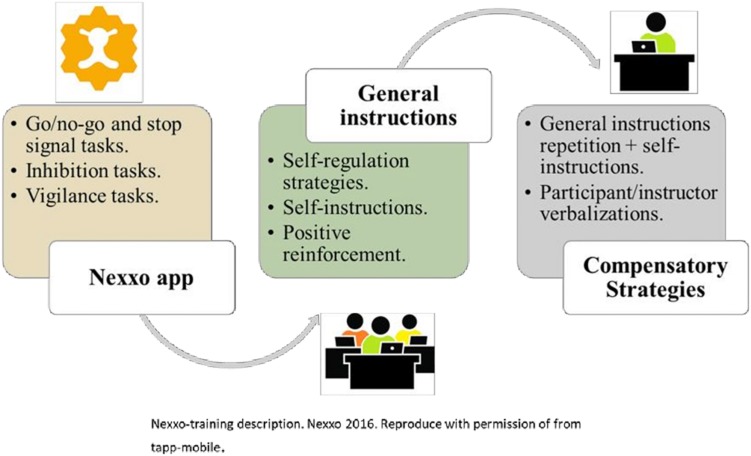
Nexxo-training.

The Nexxo-training intervention combines the repetition of EF and attentional tasks in addition to strategies to enhance the tasks. We refer to these strategies as “procedural metacognitive strategies.” In addition to general strategies aimed at the whole group, Nexxo-training provides compensatory strategies to individual participants who experience greater difficulties during training. The Nexxo application (go/no-go and stop signal tasks) was designed between 2012 and 2014, and a pilot version was developed for the study in October 2015 (Tapp-Mobile, 2015). Written informed parental consent was obtained from each participant. The participants underwent a neuropsychological assessment conducted by an examiner, which included individual tests to measure intelligence, attention, inhibition and flexibility, working memory, and processing speed. The examiners were trained psychologist who participated in the data collection. The group received a 5-week intervention conducted by a psychologist (groups of eight participants) using a special training script provided by each instructor. The Nexxo intervention was carried out over a 5-week intervention period (two sessions per week/15 min each/three games repeated twice in each session). Regarding inhibition training, a previous study of a go/no go task using a touchscreen application with preschoolers showed a trend-level improvement in reasoning and neural changes in the experimental group after 3 h of training (Liu et al., 2015). This is the reason why we decided to set the Nexxo-training duration at 3 h. The complementary strategies aimed at procedural metacognitive strategies were inspired by Perez-Hernandez and Capilla (2008). The complementary strategies were implemented by an instructor and recorded for each participant. Figure 2 shows a description of the Nexxo-training.

### Data Analysis

Statistical analyses were performed using IBM SPSS Statistics 23. Table 2 shows the frequency of participants with whom compensatory strategies were used at some point during the training. The “positive reinforcement” strategy was excluded from the following analyses because only two used it once.

**TABLE 2:** Frequency of participants with whom compensatory strategies were used at some point during the training.

	**Total *N***	**1st grade *n***	**3rd grade *n***
	**(%)**	**(%)**	**(%)**
Repeat warning to get ready	21 (45.65)	19 (67.86)	2 (11.11)
Repeat self-instructions	13 (28.26)	12 (42.86)	1 (5.56)
Instructional comprehension	35 (76.09)	25 (89.29)	10 (55.56)
Positive reinforcement	2 (4.35)	2 (7.14)	0 (0)
Child verbalizations	26 (56.52)	19 (67.86)	7 (38.89)
Instructor verbalizations	16 (34.78)	11 (39.29)	5 (27.78)
Total set of compensatory strategies	37 (80.43)	26 (92.86)	11 (61.11)

*% = percentage.*

Table 3 shows the scores in inhibition and vigilance tasks recorded by the Nexxo App, the number of total compensatory strategies applied and recorded by the instructor for children who experienced difficulties during the tasks, and the number of strategies applied of each subtype. These scores were reported for the total sample and, also, separately for the 1st and 3rd grade groups.

**TABLE 3:** Indicators of performance in inhibition and vigilance, and compensatory strategies.

	**Mean**	***SD***	**Minimum**	**Maximum**
**Inhibition**				
Total	92.5	5.93	79	100
1st grade	89.82	5.88	79	100
3rd grade	96.78	2.67	91	100
**Vigilance**				
Total	69.7	14.3	38	97
1st grade	61.79	10.73	38	85
3rd grade	82.11	9.45	60	97
**Repeat warning to get ready**				
Total	0.674	0.871	0	3
1st grade	1	0.9	0	3
3rd grade	0.17	0.51	0	2
**Repeat self-instructions**				
Total	0.609	1.42	0	8
1st grade	0.96	1.73	0	7
3rd grade	0.06	0.24	0	1
**Instructional comprehension**				
Total	2.59	2.29	0	7
1st grade	3.43	2.33	0	8
3rd grade	1.28	1.49	0	4
**Child verbalizations**				
Total	0.891	1.1	0	5
1st grade	1.18	1.25	0	5
3rd grade	0.44	0.62	0	2
**Instructor verbalizations**				
Total	0.609	1.11	0	5
1st grade	0.79	1.32	0	5
3rd grade	0.33	0.59	0	2
**Total set of compensatory strategies**				
Total	5.43	5.39	0	26
1st grade	7.46	5.81	0	26
3rd grade	2.28	2.42	0	8

*SD = standard deviation.*

For cognitive skills, we used T-scores provided by the instruments, with the exception of FDT since part of our sample was younger than the norm-based scores provided by the instrument. In this case, we calculated T-scores for our sample (1st graders and 3rd graders, separately); the higher the T-score, the lower the FDT performance.

For all the statistical analyses, the significance threshold was set at 0.05. In linear regressions, standardized β and adjusted *R*^2^ are reported.

## Results

### Compensatory Strategies and Task Performance

We used partial correlation analysis to detect the possible relation between performance and compensatory strategies, controlling for age (in months) to eliminate possible moderation due to development. After controlling for age, there was a significant correlation between inhibition and vigilance performance: the participants with a higher level of performance in inhibition games also demonstrated a higher level in vigilance games (*r* = 0.517, *p* < 0.001).

The correlations between performance in both types of tasks and compensatory strategies were significantly negative for “repeat self-instructions” and “instructional comprehension” (see Table 4), meanwhile they were marginally significant between performance in “vigilance” and “instructor verbalizations” (*r* = −0.29, *p* = 0.053). Those who obtained lower scores in the tasks (either inhibition or vigilance) required more compensatory strategies. Table 4 shows the correlations between inhibition and vigilance performance and compensatory strategies.

**TABLE 4:** Partial correlation, controlling for age in months, between performance in inhibition and vigilance, and compensatory strategies.

		**Repeat warning**	**Repeat**	**Instructional**	**Child**	**Instructor**	**Total set of**
		**to get ready**	**self-instructions**	**comprehension**	**verbalizations**	**verbalizations**	**compensatory strategies**
Inhibition	Pearson’s r	–0.229	−0.354^∗^	–0.561^∗∗∗^	–0.110	–0.256	–0.475^∗∗^
	*p*-value	0.130	0.017	<0.001	0.472	0.090	0.001
Vigilance	Pearson’s r	–0.196	−0.362^∗^	−0.342^∗^	–0.073	–0.290	–0.387^∗∗^
	*p*-value	0.197	0.014	0.022	0.635	0.053	0.009

*^∗^*p* < 0.05, ^∗∗^*p* < 0.01, and ^∗∗∗^*p* < 0.001.*

### Compensatory Strategies and Task Performance in Relation to Age

**FIGURE 3:**
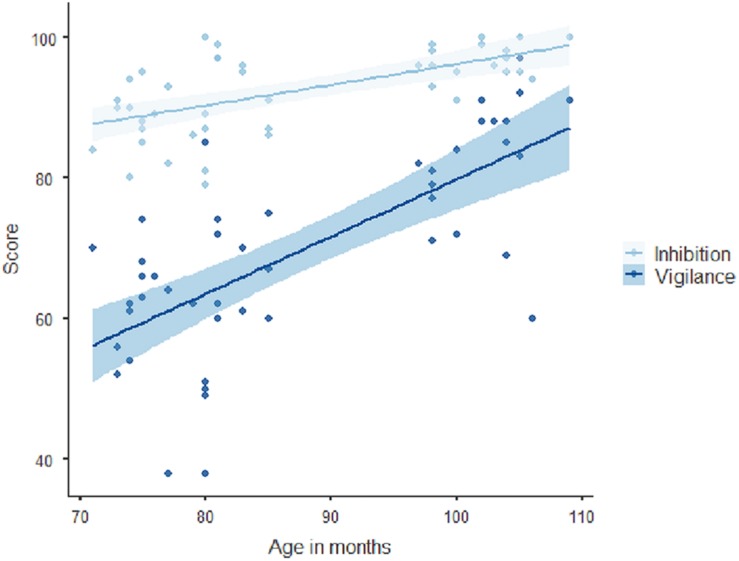
Performance in inhibition and vigilance throughout child development.

Using the participants’ age in months as an independent variable in a linear regression showed that age predicts better performance in both inhibition (β = 0.613, *p* < 0.001, adjusted *R*^2^ = 0.361) and vigilance (β = 0.706, *p* < 0.001, adjusted *R*^2^ = 0.487), with a steeper slope for vigilance: older participants have better results (see Figure 3).

Regarding the relation between age (in months) and compensatory strategies, statistically negative correlations were found with the total set of compensatory strategies, and the subtypes “repeat the warning for starting,” “instructional comprehension,” and “child verbalization” (see Table 5).

**TABLE 5:** Correlations between age in months and compensatory strategies.

		**Repeat warning**	**Repeat**	**Instructional**	**Child**	**Instructor**	**Total set of**
		**to get ready**	**self-instructions**	**comprehension**	**verbalizations**	**verbalizations**	**compensatory strategies**
Age (in months)	Pearson’s r	–0.510^∗∗∗^	–0.276	–0.484^∗∗∗^	−0.329^∗^	–0.174	–0.473^∗∗∗^
	*p*-value	<0.001	0.063	<0.001	0.026	0.248	<0.001

*^∗^*p* < 0.05, ^∗∗^*p* < 0.01, and ^∗∗∗^*p* < 0.001.*

### Cognitive Skills and Task Performance

Stepwise multiple linear regression analysis was used to identify which cognitive skills scales (DIVISA, RIST, WISC and FDT indexes) (independent variables) better predict performance in inhibition and vigilance tasks (dependent variables). For inhibition tasks, all the independent variables were non-significant. For vigilance tasks, the results showed that higher scores in odd-item-out from RIST (β = 0.389, *p* = 0.002) and lower scores in omissions from DIVISA (β = −0.479, *p* < 0.001) and flexibility from FDT (β = −0.279, *p* = 0.02) predicted better performance. Table 6 shows the complete regression model.

**TABLE 6:** Regression model predicting performance in vigilance.

	**Unstandardized**		**Standardized**	
	**coefficients**		**coefficient**	
	***B***	**SE**	**β**	***t***
Intercept	79.367	11.072	–	7.168^∗∗∗^
DIVISA-R: omissions	–0.199	0.048	–0.479	–4.417^∗∗∗^
RIST: odd-item-out	0.46	0.137	0.389	3.356^∗∗^
FDT flexibility	–0.407	0.167	–0.279	−2.431^∗^
*F*(3,38) = 13.11^∗∗∗^, adjusted *R*^2^ = 0.47				

*^∗^*p* < 0.05, ^∗∗^*p* < 0.01, and ^∗∗∗^*p* < 0.001. DIVISA-R = test of simple visual discrimination of trees – revised; RIST = reynold intellectual screening test; FDT = five digit test.*

To ascertain if this relation is present when comparing children with low and high performance in inhibition and vigilance tasks, the sample was divided into four groups using quartiles. The groups with the best performance (Q1, superior quartile) and worst performance (Q4, inferior quartile) for each task were selected for the analysis (see data in Table 7).

**TABLE 7:** Data from Q1 and Q4 groups for Inhibition and Vigilance performance.

		***n***	**Mean age**	**SD age**	**Score**	**Mean**	**SD**
			**(months)**	**(months)**		**score**	**score**
Inhibition	Q1	14 (7 M;7 F)	95.07	11.38	≥97	98.6	1.28
	Q4	11 (5 M; 6 F)	78.27	4.41	≤87	84	3
Vigilance	Q1	12 (6 M; 6 F)	101.33	7.34	≥82	87.8	4.37
	Q4	11 (4 M; 7 F)	80.82	9.15	≤60	51.6	7.85

*M = Males; F = Females; SD = standard deviation.*

Because the sample size of the groups was small, and the normality assumption was not met, a non-parametric Mann–Whitney U test was carried out to compare the differences between the Q1 and Q4 groups. Tables 8, 9 show the results for Inhibition and Vigilance, respectively.

**TABLE 8:** Mann–Whitney U test in inhibition.

	**Q1 Mdn**	**Q4 Mdn**	**Mann–Whitney U**	***p*-Value**
**DIVISA-R**				
General attention index	3	5	44.5	0.345
Commissions	85	75	55.5	0.841
Omissions	45	85	43	0.299
Organization	50	25	49	0.523
Distraction	15	10	36.5	0.131
**RIST**				
Guess what	55.5	53	51	0.153
Odd-item-out	54.5	51	70.5	0.721
General intelligence index	107.5	100	62.5	0.427
**WISC-IV**				
Symbol search	10.5	11	67	0.579
Coding	9.5	10	72.5	0.8
Digit span	12	10	54	0.201
Digit forward	11	11	56	0.229
Digit backward	12.5	12	68.5	0.639
Processing speed index	104.5	104	67.5	0.602
**FDT**				
Inhibition	45.17	53.30	52	0.171
Flexibility	45.61	50.96	49	0.134

*^∗^*p* < 0.05, ^∗∗^*p* < 0.01, and ^∗∗∗^*p* < 0.001. Mnd = median; DIVISA-R = test of simple visual discrimination of trees – revised; RIST = reynold intellectual screening test; WISC-IV = wechsler intelligence scale IV; FDT = five digit test.*

**TABLE 9:** Mann–Whitney U test in vigilance.

	**Q1 Mdn**	**Q4 Mdn**	**Mann–Whitney U**	***p*-Value**
**DIVISA-R**				
General attention index	10	2.5	28.5	0.058
Commissions	85	88	50	0.723
Omissions	20	89	22.5	0.021^∗^
Organization	35	35	46.5	0.547
Distraction	15	5	18	0.008^∗∗^
**RIST**				
Guess what	53	50	57	0.578
Odd-item-out	60	41	14.5	0.002^∗∗^
General intelligence index	113.5	91	29.5	0.024^∗^
**WISC-IV**				
Symbol search	10	8	45.5	0.201
Coding	9.5	9	49	0.283
Digit span	12	10	51	0.350
Digit forward	11	11	63	0.847
Digit backward	13	12	46.5	0.226
Processing speed index	106	96	38.5	0.09
**FDT**				
Inhibition	45.57	54.97	40	0.109
Flexibility	44.49	50.96	33	0.042^∗^

*^∗^*p* < 0.05, ^∗∗^*p* < 0.01, and ^∗∗∗^*p* < 0.001. Mnd = median; DIVISA-R = test of simple visual discrimination of trees – revised; RIST = reynold intellectual screening test; WISC-IV = wechsler intelligence scale IV; FDT = five digit test.*

Concerning inhibition tasks, no differences were found between the Q1 and Q4 groups in any of the skills assessed. Nevertheless, for vigilance tasks, the scores were significantly higher for Q1 in distraction from DIVISA (*U* = 18, *p* = 0.008), odd-item-out subtest (*U* = 14.5, *p* = 0.002) and general index (*U* = 29.5, *p* = 0.024) from RIST, and lower in omissions from DIVISA (*U* = 22.5, *p* = 0.021) and flexibility from FDT (*U* = 33, *p* = 0.042).

## Discussion

Nexxo-training is an innovative strategy-based training for attention and EF. Strategy-based training combines the repetition of a task with strategies (e.g., scaffolding or metacognitive strategies) to improve performance (Morrison and Chein, 2011; Jolles and Crone, 2012). In this study, the Nexxo-training involved computer-based training through “go/no-go” and “stop signal” tasks, in combination with procedural metacognitive strategies for the whole group, adapted to the participants’ developmental stage, as well as compensatory strategies for those who presented greater difficulties during the training. The tasks were developed using an application (“Nexxo” iPad application). As touchscreens and applications are appealing to children (Lai et al., 2013), this approach can motivate them to participate in the training. This new training approach has demonstrated positive results in school-age students in terms of attention and EF (Rossignoli-Palomeque et al., unpublished). To our knowledge, this is the first (strategy-based) cognitive training that provides, compensatory strategies for participants who experience greater difficulties. Considering the proportion of participants who required compensatory strategies at some point in the training period (80.43%), it seems that compensatory strategies are relevant over the course of the training process. The most commonly used compensatory strategy was instructional comprehension (76.05%), followed by child verbalizations (56.52%), repeating warning for starting (45.65%), instructor verbalizations (34.78%), repeating of self-instructions (28.26%), and gestures reinforcement (4.35%). Instructional comprehension (i.e., verbalizations of what to do) was the strategy most commonly required by both 1st-grade and 3rd-grade participants. This strategy is fundamental in self-regulated learning (Dina and Efklides, 2009). As shown in Table 2, the younger participants displayed a greater need for repeating instructions to get ready (67.86% in 1st grade vs. 11.11% in 3rd grade), child verbalizations (67.86% in 1st grade vs. 38.89% in 3rd grade), and self-instructions (42.86% in 1st grade vs. 5.56% in 3rd grade). These results may be due to a greater development of attentional control and inner speech around the 3rd grade. As suggested by Winsler et al. (2009), inner speech evolves from irrelevant speech to self-directed verbalizations that are relevant for the task. Strategy-based attention and EF training with compensatory strategies is a new direction, and further research on attention and EF training should focus on strategies that are more likely to improve task performance and far transfer. Indeed, it is crucial to conduct this type of training research on strategies used by students while performing attention and EF tasks.

Cognitive training should be designed based on neuropsychological models. The Nexxo application is founded on well-known attention and EF paradigms (Shiffrin and Schneider, 1977; Logan, 1994). In addition, the strategies, self-regulation strategies (motor and verbal strategies), instructional comprehension, and self-instruction have been designed considering developmental factors (Vygotsky et al., 1978; Bjorklund and Harnishfeger, 1990). As reviewed in scientific literature, verbal and motor strategies are used by preschoolers to inhibit themselves (Fatzer and Roebers, 2013; Manfra et al., 2014), and internal verbalizations evolve from irrelevant speech (at 2 years of age) to self-directed instructions that are relevant to the tasks (at 8 years of age) (Winsler et al., 2009). Thus, it seems reasonable to use self-directed instructions as a verbal strategy in school-age students in combination with motor strategies for self-control. Finally, Nexxo-training also involves procedural metacognitive strategies, such as self-instruction and instructional comprehension strategies, to promote self-control and attention. As cognition and self-regulation are viewed as an integral unit (Vygotsky et al., 1978), by combining computer-based training in attention and EF with procedural metacognitive strategies selected for the appropriate developmental period, the training will help to improve these processes as they develop naturally. This should be the criteria when selecting the training strategies. Teaching children to control their own behavior can lead to more durable behavioral changes and less dependency on adult supervision (O’Leary and Dubey, 1979). The student’s use of procedural metacognitive strategies, such as selection, monitoring, and control of their learning activities, is crucial for their achievement in all learning situations (Zimmerman, 2011). This can be justified by the theoretical overlap between EF and procedural metacognition (Roebers and Feurer, 2016). For this reason, we consider that analyzing strategy-based training is relevant for the increased likelihood of transference and long-term effects. Finally, cognitive training researchers should consider studying strategies that can be applied in attention and EF training at different developmental stages.

In this study, we analyzed the compensatory strategies used by participants experiencing difficulties in EF and attention tasks. In addition, we analyzed the developmental factors and cognitive skills that may modulate EF and attention task performance. This is relevant for the future of attention and EF cognitive training design. First, we found a positive correlation between inhibition and vigilance. This result is supported by previous findings suggesting a relation between the two elements (Lovejoy and Rasmussen, 1990; Corbetta and Shulman, 2002; Friedman and Miyake, 2004; Rebollo and Montiel, 2006; Tirapu Ustárroz, 2012). As inhibition is central to EF (Dempster, 1992), and vigilance is central to attention (Hauke et al., 2011), we believe that the combination of both processes may help to improve more complex subcomponents of attention and EF. The results are consistent with previous findings that connect attention and EF (Lovejoy and Rasmussen, 1990; Pérez-Edgar et al., 2010).

Regarding the procedural metacognitive strategies used during task performance, our analysis showed that those who obtained lower scores in task performance (either inhibition or vigilance) required more compensatory strategies. Compensatory strategies provide a way for participants to adapt to the training. Specifically, the participants with lower inhibition and vigilance scores in the application required more instructional comprehension as a compensatory strategy. Similarly, those with lower task performance and a higher number of omissions in the DIVISA-R test (Santacreu et al., 2010), which is related to inattention, depended more on the instructional comprehension strategy. As mentioned above, instructional comprehension and self-instruction strategy can help participants to establish a goal, plan and monitor task performance (Dina and Efklides, 2009). Moreover, repeating instructions helps to overcome difficulties in working memory (Baddeley, 1992). This finding is robust considering the effectiveness that self-instruction has shown in students with difficulties in attention and EF, such as ADHD (Harris et al., 2004; Gawrilow and Gollwitzer, 2008). For these participants, repeating instructions using self-instruction and goal setting was fundamental. Future strategy-based training designs for attention and EF should consider these findings.

One of the objectives of the study was to analyze the influence of age in task performance in order to identify the appropriate age for Nexxo-training. As hypothesized, the older participants obtained better results in inhibition and vigilance tasks; therefore, age moderates task performance. This may be due to neuropsychological changes that occur during child development (Duncan and Owen, 2000; Collette et al., 2005). In terms of inhibition performance using go/no-go tasks for assessment, it seems that there is an improvement in response inhibition abilities moderated by age (Becker et al., 1987; Lewis et al., 2017a), which makes this period relevant. In this regard, our finding is consistent with previous scientific literature. Furthermore, age moderates the use of strategies, as statically negative correlations were found with the total set of compensatory strategies, and the subtypes (“repeat the warning for starting,” “comprehension instructions,” and “child verbalization”). This finding is consistent with the progressive development of verbal strategies and self-instruction (Vygotsky et al., 1978; Bjorklund and Harnishfeger, 1990). According to these findings, and, consistent with our results, using this type of training with children up to the age of 8 years old seems ideal.

Regarding cognitive skills and task performance, our results shows that higher scores in RIST odd-item-out (fluid intelligence), and lower levels of Omissions in DIVISA (attention test) and in FDT flexibility (cognitive flexibility) predicts better results in vigilance tasks. Recent research shows that working memory, inhibition and shifting, the main components of EF, contribute substantially to general intellectual ability, especially fluid intelligence (Chen et al., 2019). Meanwhile, the parietal and frontal areas involved in EF have also been related to fluid intelligence (Tschentscher et al., 2017; Yoon et al., 2017). Consequently, based on this idea, we analyzed the relation between inhibition and vigilance task performance with fluid intelligence. Our results show that fluid intelligence predicts better results in vigilance. Vigilance tasks require attentional control which is related to inhibitory control. We also found that participants with higher levels of performance in vigilance also obtained higher scores in fluid intelligence. Previous findings have suggested a relation between vigilance and intelligence in children at risk of learning disabilities (Swanson and Cooney, 1989). In this sense, we must add that intelligence benefits vigilance performance. In terms of attention, our results show that the participants with fewer omissions and a lower level of distractibility in neuropsychological tests had better results in vigilance task (after training). As demonstrated in previous studies, omissions and distractibility can be predictors of go/no-go performance (Lewis et al., 2017b). In our view, the fact that lower levels of omissions in the DIVISA-R test is related to better performance in vigilance, is a result which provides validity to the training. Finally, as regards the relation between cognitive flexibility and attention, we consider that cognitive flexibility has a positive influence on vigilance tasks as the instructions change for each game. The transition from one rule (e.g., “tap each time a bear appears on the screen) to another (e.g., “tap when you see the number 5”) involves not only an alteration in the type of instructions (target and distractors) but also a change from vigilance tasks to inhibition tasks, as both types of games are played in each session. We hypothesize that individuals with higher cognitive flexibility may better adjust their cognitive resources to these changes. A previous study suggested that cognitive flexibility may become a useful tool for vigilance training strategies, as individual differences in cognitive flexibility predicts better results in vigilance tasks (Figueroa and Youmans, 2012). Another possible explanation refers to the idea of flexibility as a predictor of response speed (Deák and Wiseheart, 2015). Go/no-go tasks involve response speed, i.e., a participant with a low response speed may produce a high number of omissions in the task and, as a result, obtain lower levels of vigilance performance. All these examples demonstrate how cognitive processes are interrelated, and, therefore, how training may have a simultaneous impact on multiple processes.

This study has several key strengths. Firstly, it examines a type of strategy-based training in attention and EF functions that provides compensatory strategies adapted to the participant’s needs. This is an innovative approach for cognitive training with potential for further research. Secondly, the cognitive training tasks presented in the Nexxo app are based on neuropsychological models (Shiffrin and Schneider, 1977; Logan, 1994). Furthermore, the implemented strategies are based on previous research and have been designed according to the developmental stage at which the training is applied. In this regard, it is important for future strategy-based training designs to consider child developmental factors. In our view, this approach can overcome the limitations of previous cognitive training designs in attention and EF, in terms of generalization and long-term effects (Rossignoli-Palomeque et al., 2018). Thirdly, this analysis has helped to clarify the relevance of instructional comprehension and self-instruction as compensatory strategies. This finding should also be taken into consideration for future training designs. This study reveals that child development moderates inhibition and vigilance performance. In addition, this paper demonstrates that there is a relation between fluid intelligence and vigilance. This finding raises the question of whether intelligence can be improved by training vigilance. However, further research is needed in this area. In addition, our paper shows a relation between inhibition and vigilance. Nevertheless, this study also had certain limitations. For example, as the study did not involve groups of older participants, we could not analyze the feasibility of the strategies in different age groups. In addition, due to a technical limitation, we were unable to include processing speed as a variable in our analysis. Therefore, it would be advantageous to include this variable in future training designs.

Finally, we focused on Nexxo-training with typically developing children. Further research on Nexxo-training should focus on atypically developing children in terms of attention and EF, such as ADHD.

## Conclusion

Nexxo-training is a specific form of strategy-based training that provides not only general procedural metacognitive strategies for the whole group, but also compensatory strategies for individual participants who experience greater difficulties during the training. Considering the proportion of participants who required compensatory strategies at some point in the training period (80.43%), it seems that compensatory strategies are relevant over the course of the training process. Regarding strategy analysis, instructional comprehension and self-instruction (e.g., goal setting and planning) seem to be the most useful strategies for participants with difficulties in inhibitory and vigilance task performance. Finally, developmental factors moderate task performance, while fluid intelligence and cognitive flexibility is related to vigilance performance.

## Data Availability Statement

All datasets generated for this study are included in the manuscript/Supplementary Files.

## Ethics Statement

In accordance with the Declaration of Helsinki, written informed parental consent was obtained from each participant. This study was approved by the Ethics Committee of the San Carlos Hospital (*n*°186; 15/315-E) in June 2015.

## Author Contributions

TR-P conceived the application. TR-P, EP-H, and JG-M conceived, design, and coordinated the experiment. MQ-G involved in the data analysis. TR-P and MQ-G wrote the manuscript. EP-H reviewed the manuscript.

## Conflict of Interest

TR-P owns the original idea for the Nexxo application and participated in the design of the Nexxo games. The Nexxo application for iPad is a commercially available app (a free-to-install app with in-app purchases). TR-P is part of the development team for the Nexxo application for iPad.

The remaining authors declare that the research was conducted in the absence of any commercial or financial relationships that could be construed as a potential conflict of interest.

## References

[B1] Andersson U. (2008). Working memory as a predictor of written arithmetical skills in children: the importance of central executive functions. *Br. J. Educ. Psychol.* 78 181–203. 10.1348/000709907x209854 17535520

[B2] Baddeley A. (1992). Working memory. *Science* 255 556–559. 1736359 10.1126/science.1736359

[B3] Barkley R. A. (1997). Behavioral inhibition, sustained attention, and executive functions: constructing a unifying theory of ADHD. *Psychol. Bull.* 121 65–94. 10.1037/0033-2909.121.1.65 9000892

[B4] Beck S. J. Hanson C. A. Puffenberger S. S. Benninger K. L. Benninger W. B. (2010). A controlled trial of working memory training for children and adolescents with ADHD. *J. Clin. Child Adolesc. Psychol.* 39 825–836. 10.1080/15374416.2010.517162 21058129

[B5] Becker M. G. Isaac W. Hynd G. W. (1987). Neuropsychological development of nonverbal behaviors attributed to “frontal lobe” functioning. *Dev. Neuropsychol.* 3 275–298. 10.1080/87565648709540381

[B6] Bjorklund D. F. Harnishfeger K. K. (1990). “Children’s strategies: their definitions and origins,” in *Children’s Strategies: Contemporary Views of Cognitive Development*, ed. Bjorklund D. F., (Hillsdale, NJ: Lawrence Erlbaum Associates, Inc), 309–323.

[B7] Blair C. Razza R. P. (2007). Relating effortful control, executive function, and false belief understanding to emerging math and literacy ability in kindergarten. *Child Dev.* 78 647–663. 10.1111/j.1467-8624.2007.01019.x 17381795

[B8] Blakemore S. Choudhury S. (2006). Development of the adolescent brain: implications for executive function and social cognition. *J. Child Psychol. Psychiatry* 47 296–312. 10.1111/j.1469-7610.2006.01611.x 16492261

[B9] Bryck R. L. Fisher P. A. (2012). Training the brain: practical applications of neural plasticity from the intersection of cognitive neuroscience, developmental psychology, and prevention science. *Am. Psychol.* 67 87–100. 10.1037/a0024657 21787037 PMC3335430

[B10] Carlson S. M. White R. E. (2013). “Executive function, pretend play, and imagination,” in *The Oxford Handbook of the Development of Imagination*, ed. Taylor M. (New York: Oxford University Press), 161–174.

[B11] Chen Y. Spagna A. Wu T. Kim T. H. Wu Q. Chen C. (2019). Testing a cognitive control model of human intelligence. *Sci. Rep.* 9:2898. 10.1038/s41598-019-39685-2 30814663 PMC6393508

[B12] Ciesielski K. Harris R. J. (1997). Factors related to performance failure on executive tasks in autism. *Child Neuropsychol.* 3 1–12. 10.1080/09297049708401364

[B13] Collette F. Van der Linden M. Laureys S. Delfiore G. Degueldre C. Luxen A. (2005). Exploring the unity and diversity of the neural substrates of executive functioning. *Hum. Brain Mapp.* 25 409–423. 10.1002/hbm.20118 15852470 PMC6871684

[B14] Conway A. R. Kane M. J. Engle R. W. (2003). Working memory capacity and its relation to general intelligence. *Trends Cogn. Sci.* 7 547–552. 10.1016/j.tics.2003.10.005 14643371

[B15] Corbetta M. Shulman G. L. (2002). Control of goal-directed and stimulus-driven attention in the brain. *Nat. Rev. Neurosci.* 3 201–215. 10.1038/nrn755 11994752

[B16] Crick N. R. Dodge K. A. (1994). A review and reformulation of social information-processing mechanisms in children’s social adjustment. *Psychol. Bull.* 115 74–101. 10.1093/deafed/enw030 27143715

[B17] Dahlin K. I. (2011). Effects of working memory training on reading in children with special needs. *Read. Writ.* 24 479–491. 10.1016/j.ridd.2014.08.013 25200678

[B18] Dahlin K. I. (2013). Working memory training and the effect on mathematical achievement in children with attention deficits and special needs. *J. Educ. Learn.* 2 118–133.

[B19] Deák G. O. Wiseheart M. (2015). Cognitive flexibility in young children: general or task-specific capacity? *J. Exp. Child Psychol.* 138 31–53. 10.1016/j.jecp.2015.04.003 26026421

[B20] Dempster F. N. (1992). The rise and fall of the inhibitory mechanism: toward a unified theory of cognitive development and aging. *Dev. Rev.* 12 45–75. 10.1016/0273-2297(92)90003-k

[B21] Diamond A. (2013). Executive functions. *Annu. Rev. Psychol.* 64 135–168. 10.1146/annurev-psych-113011-143750 23020641 PMC4084861

[B22] Diamond A. Barnett W. S. Thomas J. Munro S. (2007). Preschool program improves cognitive control. *Science* 318 1387–1388. 10.1126/science.1151148 18048670 PMC2174918

[B23] Dina F. Efklides A. (2009). “Motivation and affect in self-regulated learning: does metacognition play a role?,” in *Educational Psychology Handbook Series. Handbook of Self-regulation of Learning and Performance*, eds Schunk D. H. Greene J. A., (New York, NY: Routledge/Taylor & Francis Group), 64–82. 10.4324/9781315697048-5

[B24] Duncan J. Owen A. M. (2000). Common regions of the human frontal lobe recruited by diverse cognitive demands. *Trends Neurosci.* 23 475–483. 10.1016/s0166-2236(00)01633-7 11006464

[B25] Enright S. J. Beech A. R. (1993). Further evidence of reduced cognitive inhibition in obsessive-compulsive disorder. *Personal. Individ. Differ.* 14 387–395. 10.1016/0191-8869(93)90307-o 8467275

[B26] Fatzer S. Roebers C. (2013). Language and executive functioning: children’s benefit from induced verbal strategies in different tasks. *J. Educ. Dev. Psychol.* 3 1–9.

[B27] Fernyhough C. Fradley E. (2005). Private speech on an executive task: relations with task difficulty and task performance. *Cogn. Dev.* 20 103–120. 10.1016/j.cogdev.2004.11.002

[B28] Figueroa I. J. Youmans R. J. (2012). Individual differences in cognitive flexibility predict performance in vigilance tasks. *Pap. Present. Proc. Hum. Factors and Ergon. Soc. Ann. Meet.* 56 1099–1103. 10.1177/1071181312561239

[B29] Flores-Lázaro J. C. Castillo-Preciado R. E. Jiménez-Miramonte N. A. (2014). Desarrollo de funciones ejecutivas, de la niñez a la juventud. *Anales de Psicologia* 30 463–473. 10.6018/analesps.30.2.155471

[B30] Friedman N. P. Miyake A. (2004). The relations among inhibition and interference control functions: a latent-variable analysis. *J. Exp. Psychol.* 133 101–135. 10.1037/0096-3445.133.1.101 14979754

[B31] Gandolfi E. Viterbori P. Traverso L. Usai M. C. (2014). Inhibitory processes in toddlers: a latent-variable approach. *Front. Psychol.* 5:381. 10.3389/fpsyg.2014.00381 24817858 PMC4012180

[B32] Garon N. Bryson S. E. Smith I. M. (2008). Executive function in preschoolers: a review using an integrative framework. *Psychol. Bull.* 134 31–60. 10.1037/0033-2909.134.1.31 18193994

[B33] Gathercole S. E. Pickering S. J. Ambridge B. Wearing H. (2004). The structure of working memory from 4 to 15 years of age. *Dev. Psychol.* 40 177–190. 10.1037/0012-1649.40.2.177 14979759

[B34] Gau S. S. Lin Y. J. Cheng A. T. Chiu Y. N. Tsai W. C. Soong W. T. (2010). Psychopathology and symptom remission at adolescence among children with attention-deficit-hyperactivity disorder. *Aust. N. Z. J. Psychiatry* 44 323–332. 10.3109/00048670903487233 20307165

[B35] Gawrilow C. Gollwitzer P. M. (2008). Implementation intentions facilitate response inhibition in children with ADHD. *Cogn. Ther. Res.* 32 261–280. 10.1007/s10608-007-9150-1

[B36] Harris K. Reid R. R. Graham S. (2004). “Self-Regulation among Students with LD and ADHD,” in *Learning About Learning Disabilities*, ed. Wong B. (Amsterdam: Elsevier Inc), 167–195. 10.1016/b978-012762533-1/50008-1

[B37] Hauke J. Fimm B. Sturm W. (2011). Efficacy of alertness training in a case of brainstem encephalitis: clinical and theoretical implications. *Neuropsychol. Rehabil.* 21 164–182. 10.1080/09602011.2010.541792 21391120

[B38] Holmes J. Gathercole S. E. (2014). Taking working memory training from the laboratory into schools. *Educ. Psychol.* 34 440–450. 10.1080/01443410.2013.797338 26494933 PMC4579053

[B39] Jolles D. Crone E. A. (2012). Training the developing brain: a neurocognitive perspective. *Front. Hum. Neurosci.* 6:76. 10.3389/fnhum.2012.00076 22509161 PMC3321411

[B40] Kaplan S. Berman M. G. (2010). Directed attention as a common resource for executive functioning and self-regulation. *Perspect. Psychol. Sci.* 5 43–57. 10.1177/1745691609356784 26162062

[B41] Karbach J. Kray J. (2009). How useful is executive control training? Age differences in near and far transfer of task−switching training. *Dev. Sci.* 12 978–990. 10.1111/j.1467-7687.2009.00846.x 19840052

[B42] Karbach J. Unger K. (2014). Executive control training from middle childhood to adolescence. *Front. Psychol.* 5:390. 10.3389/fpsyg.2014.00390 24847294 PMC4019883

[B43] Klingberg T. Fernell E. Olesen P. J. Johnson M. Gustafsson P. Dahlström K. (2005). Computerized training of working memory in children with ADHD-a randomized, controlled trial. *J. Am. Acad. Child Adolesc. Psychiatry* 44 177–186. 10.1097/00004583-200502000-00010 15689731

[B44] Klingberg T. Forssberg H. Westerberg H. (2002). Training of working memory in children with ADHD. *J. Clin. Exp. Neuropsychol.* 24 781–791. 12424652 10.1076/jcen.24.6.781.8395

[B45] La Marca J. P. O’Connor R. E. (2016). Neurofeedback as an intervention to improve reading achievement in students with attention deficit hyperactivity disorder, inattentive subtype. *NeuroRegulation* 3 55–77. 10.15540/nr.3.2.55

[B46] Lai K. Khaddage F. Knezek G. (2013). Blending student technology experiences in formal and informal learning. *J. Comput. Assisted Learn.* 29 414–425. 10.1111/jcal.12030

[B47] Lewis F. C. Reeve R. A. Kelly S. P. Johnson K. A. (2017a). Evidence of substantial development of inhibitory control and sustained attention between 6 and 8 years of age on an unpredictable go/no-go task. *J. Exp. Child Psychol.* 157 66–80. 10.1016/j.jecp.2016.12.008 28119117

[B48] Lewis F. C. Reeve R. A. Kelly S. P. Johnson K. A. (2017b). Sustained attention to a predictable, unengaging go/no-go task shows ongoing development between 6 and 11 years. *Atten. Percept. Psychophys.* 79 1726–1741. 10.3758/s13414-017-1351-4 28597322

[B49] Liu Q. Zhu X. Ziegler A. Shi J. (2015). The effects of inhibitory control training for preschoolers on reasoning ability and neural activity. *Sci. Rep.* 5:14200. 10.1038/srep14200 26395158 PMC4585799

[B50] Logan G. D. (1994). “On the ability to inhibit thought and action: a users’ guide to the stop signal paradigm,” in *Inhibitory Processes in Attention, Memory, and Language*, eds Dagenbach D. Carr T. H., (San Diego, CA: Academic Press), 189–239.

[B51] Lovejoy M. C. Rasmussen N. H. (1990). The validity of vigilance tasks in differential diagnosis of children referred for attention and learning problems. *J. Abnorm. Child Psychol.* 18 671–681. 10.1007/bf01342753 2074345

[B52] Manfra L. Davis K. D. Ducenne L. Winsler A. (2014). Preschoolers’ motor and verbal self-control strategies during a resistance-to-temptation task. *J. Genetic Psychol.* 175 332–345. 10.1080/00221325.2014.917067 25175682

[B53] Miyake A. Friedman N. P. Emerson M. J. Witzki A. H. Howerter A. Wager T. D. (2000). The unity and diversity of executive functions and their contributions to complex “frontal lobe” tasks: a latent variable analysis. *Cogn. Psychol.* 41 49–100. 10.1006/cogp.1999.0734 10945922

[B54] Molfese V. J. Molfese P. J. Molfese D. L. Rudasill K. M. Armstrong N. Starkey G. (2010). Executive function skills of 6–8-year-old: brain and behavioral evidence and implications for school achievement. *Contemp. Educ. Psychol.* 35 116–125. 10.1016/j.cedpsych.2010.03.004 20798857 PMC2925418

[B55] Morrison A. B. Chein J. M. (2011). Does working memory training work? The promise and challenges of enhancing cognition by training working memory. *Psychon. Bull. Rev.* 18 46–60. 10.3758/s13423-010-0034-0 21327348

[B56] O’Leary S. G. Dubey D. R. (1979). Applications of self−control procedures by children: a review. *J. Appl. Behav. Anal.* 12 449–465. 10.1901/jaba.1979.12-449 389917 PMC1311430

[B57] Olson S. L. (1989). Assessment of impulsivity in preschoolers: cross-measure convergences, longitudinal stability, and relevance to social competence. *J. Clin. Child Psychol.* 18 176–183. 10.1207/s15374424jccp1802_9

[B58] Pérez-Edgar K. McDermott J. N. M. Korelitz K. Degnan K. A. Curby T. W. Pine D. S. (2010). Patterns of sustained attention in infancy shape the developmental trajectory of social behavior from toddlerhood through adolescence. *Dev. Psychol.* 46 1723–1730. 10.1037/a0021064 20873921 PMC3756607

[B59] Perez-Hernandez E. Capilla A. (2008). “Neuropsicologia infantil,” in *Manual de Neuropsicologia*, eds Tirapu Ustárroz J. Ríos Lago M. Maestu F. Viguera:Barcelona, 447–474.

[B60] Pontifex M. B. Scudder M. R. Drollette E. S. Hillman C. H. (2012). Fit and vigilant: the relationship between poorer aerobic fitness and failures in sustained attention during preadolescence. *Neuropsychology* 26 407–413. 10.1037/a0028795 22746307 PMC3390762

[B61] Pozuelos J. P. Combita L. M. Abundis A. Paz−Alonso P. M. Conejero Á Guerra S. (2018). Metacognitive scaffolding boosts cognitive and neural benefits following executive attention training in children. *Dev. Sci.* 22 e12756. 10.1111/desc.12756 30257077

[B62] Pozuelos J. P. Paz-Alonso P. M. Castillo A. Fuentes L. J. Rueda M. R. (2014). Development of attention networks and their interactions in childhood. *Dev. Psychol.* 50 2405–2415. 10.1037/a0037469 25069052

[B63] Rebollo M. A. Montiel S. (2006). Atención y funciones ejecutivas. *Rev. Neurol.* 42 S3–S7. 16555217

[B64] Reck S. G. Hund A. M. (2011). Sustained attention and age predict inhibitory control during early childhood. *J. Exp. Child Psychol.* 108 504–512. 10.1016/j.jecp.2010.07.010 20801457

[B65] Reynolds C. R. Kamphaus R. W. (2003). *RIST Reynolds Intellectual Screening test. Interpretative manual.* Torrance, CA: Western Psychological Services.

[B66] Roebers C. M. Feurer E. (2016). Linking executive functions and procedural metacognition. *Child Dev. Perspect.* 10 39–44. 10.1111/cdep.12159

[B67] Rossignoli-Palomeque T. Perez-Hernandez E. González Marqués J. (2018). Brain training in children and adolescents: is it scientifically valid? *Front. Psychol.* 9:565. 10.3389/fpsyg.2018.00565 29780336 PMC5946581

[B68] Rothbart M. K. Posner M. I. (2001). “Mechanism and variation in the development of attentional networks,” in *Handbook of Developmental Cognitive Neuroscience*, eds Nelson C. A. Luciana M., (Cambridge, MA: MIT Press), 353–363.

[B69] Rueda M. R. Checa P. Cómbita L. M. (2012). Enhanced efficiency of the executive attention network after training in preschool children: immediate changes and effects after two months. *Dev. Cog. Neurosci.* 2(Suppl 1), S192–S204. 10.1016/j.dcn.2011.09.004 22682908 PMC6987678

[B70] Rueda M. R. Posner M. I. Rothbart M. K. (2005). The development of executive attention: contributions to the emergence of self-regulation. *Dev. Neuropsychol.* 28 573–594. 10.1207/s15326942dn2802_2 16144428

[B71] Santacreu J. Shih P. Quiroga M. (2010). *DIVISA, Test de Discriminación Visual Simple de árboles [DIVISA, Trees Simple Visual Discrimination Test].* Madrid: TEA Ediciones.

[B72] Sastre-Riba S. Viana-Sáenz L. (2016). Funciones ejecutivas y alta capacidad intelectual. *Rev. Neurol.* 62 s65–s71. 26922961

[B73] Sedó M. A. (2007). *FDT: Test de los Cinco Dígitos.* Madrid: TEA Ediciones.

[B74] Shiffrin R. M. Schneider W. (1977). Controlled and automatic human information processing: II. perceptual learning, automatic attending and a general theory. *Psychol. Rev.* 84 127–190.

[B75] Sturm W. (2008). “Aufmerksamkeit (attention),” in *Handbuch Der Neuro-Und Biopsychologie*, eds Gauggle S. Herrmann M., (Gottingen: Hogrefe), 329–336.

[B76] Sturm W. Willmes K. (2001). On the functional neuroanatomy of intrinsic and phasic alertness. *NeuroImage* 14 S76–S84. 11373136 10.1006/nimg.2001.0839

[B77] Swanson H. L. Cooney J. B. (1989). Relationship between intelligence and vigilance in children. *J. Sch. Psychol.* 27 141–153. 10.1016/0022-4405(89)90002-2

[B78] Tamm L. Nakonezny P. A. (2015). Metacognitive executive function training for young children with ADHD: a proof-of-concept study. *ADHD Atten. Defic. Hyperact. Disord.* 7 183–190. 10.1007/s12402-014-0162-x 25559877 PMC4492907

[B79] Tapp-Mobile (2015). *Nexxo (version 1.0) [Touchscreen Application].* Available at: https://apps.apple.com/es/app/nexxo/id979045960. 10.1007/s12402-014-0162-x 25559877 PMC4492907

[B80] Thorell L. B. Lindqvist S. Bergman Nutley S. Bohlin G. Klingberg T. (2009). Training and transfer effects of executive functions in preschool children. *Dev. Sci.* 12 106–113. 10.1111/j.1467-7687.2008.00745.x 19120418

[B81] Tirapu Ustárroz J. (2012). *Neuropsicología de la Corteza Prefrontal y las Funciones ejecutivas.* Barcelona: Viguera.

[B82] Tschentscher N. Mitchell D. Duncan J. (2017). Fluid intelligence predicts novel rule implementation in a distributed frontoparietal control network. *J. Neurosci.* 37 4841–4847. 10.1523/JNEUROSCI.2478-16.2017 28408412 PMC5426573

[B83] Tsujimoto S. Kuwajima M. Sawaguchi T. (2007). Developmental fractionation of working memory and response inhibition during childhood. *Exp. Psychol.* 54 30–37. 10.1027/1618-3169.54.1.30 17341012

[B84] Urben S. van der Linden M. Barisnikov K. (2011). Development of the ability to inhibit a prepotent response: influence of working memory and processing speed. *Br. J. Dev. Psychol.* 29 981–998. 10.1111/j.2044-835X.2011.02037.x 21995748

[B85] van der Ven S. H. G. Kroesbergen E. H. Boom J. Leseman P. P. M. (2013). The structure of executive functions in children: a closer examination of inhibition, shifting, and updating. *Br. J. Dev. Psychol.* 31 70–87. 10.1111/j.2044-835X.2012.02079.x 23331107

[B86] Vygotsky L. S. Cole M. John-Steiner V. Scribner S. Souberman E. (eds) (1978). “The development of higher psychological processes,” in *Mind in Society*, (Cambridge, MA: Harvard University Press), 1–91.

[B87] Wechsler D. (2005). in *WISC-IV: Escala de Inteligencia de Wechsler para Niños-IV*, eds Corral S. Arribas D. Santamaría P. Suerio M. J. Peñera Y. J., (Madrid: TEA ediciones).

[B88] Winsler A. E. Fernyhough C. E. Montero I. E. (2009). *Private Speech, Executive Functioning, and the Development of Verbal self-Regulation.* Cambridge, MA: Cambridge University Press.

[B89] Yoon Y. B. Shin W. Lee T. Y. Hur J. Cho K. I. K. Sohn W. S. (2017). Brain structural networks associated with intelligence and visuomotor ability. *Sci. Rep.* 7:2177. 10.1038/s41598-017-02304-z 28526888 PMC5438383

[B90] Zelazo P. D. Müller U. (2002). Executive function in typical and atypical development. *Blackwell Handb. Child. Cogn. Dev.*, 445–469. 10.1002/9780470996652.ch20

[B91] Zimmerman B. J. (2011). “Motivational sources and outcomes of self-regulated learning and performance,” in *Handbook of Self-Regulation of Learning and Performance*, eds Zimmerman B. J. Schunk D. H., (New York, NY: Routledge/Taylor & Francis Group), 49–64.

