# Supplementary material for: Schoolchildren’s Compensatory Strategies and Skills in Relation to Attention and Executive Function App Training
Source: Front Psychol. 2019 Oct 15;10:2332. doi: 10.3389/fpsyg.2019.02332 (PMC6843073; doi:10.3389/fpsyg.2019.02332)
Supplement: Supplementary file 1 [file Data_Sheet_1.docx]

Supplementary Material

Table 1

*Procedural metacognitive strategies applied in Nexxo-training*

| Type |  | Description |  |
| --- | --- | --- | --- |
| First day general instructions (instructor’s commands). |  | Presentation:  - “We are going to enter a room with a touchscreen, you have to sit where I tell you. The touchscreen cannot be touched until I say so”.  - “My name is (instructor’s name). We are a team. We will have 10 sessions to play a game”.  Rules:  a) You will use the same touchscreen each day and I will tell you in which game you need to put on the headphones.  b) You can see some stickers on the table. You should put your hands on them when I say, “in position”.  d) You also can see an image of a clock-eye-hand on the table (wait-see-tap). It’s a reminder of how to play.  e) I will tell you if you have to press “V” or “I” block on the application, and I will read the game’s instructions aloud.  f) Each time you play, when the screen turns red, it means you are wrong, whereas if it’s green, it means you are doing great. When you finish each game, you will see how many stars you got.  g) We have to be good observers and not fall into the traps.  h) We will play each game twice to get better results. |  |
| 1. General instructions |  | a) Signal to prepare for the start of the session “in position”.  b) “Visual self-instruction” (wait-see-tap).  c) Verbal self-instructions: “I am a good observer, I will not fall into the traps”.  d) Instructions comprehension. Instructor´s specific command clarification. “When do we have to tap?”.  e) Verbal reinforcement after games. |  |
| 2. Compensatory strategies |  | a) Repeating the signal for starting (“in position”).  b) Repeating self-instruction (wait-see-tap).  c) Repeating instructions.  d) Child verbalizations through the game (say out loud what appears on the screen).  e) Instructor verbalizations (say out loud what appears on the screen).  f) Positive reinforcement through gestures or saying “well done” out loud. |  |

*Note.* Procedural metacognitive strategies inspired by Pérez-Hernandez et al., (2011). First day general instructions: applied to the whole group in the first session. 1. General instructions: applied each session to the whole group. 2 Compensatory strategies: applied to those who presented difficulties while training.
